# Identification and validation of disease severity-related circular RNA in acute pancreatitis: Disease severity-related circRNA in acute pancreatitis

**DOI:** 10.3724/abbs.2024115

**Published:** 2024-09-25

**Authors:** Jiarong Li, Zefang Sun, Caihong Ning, Chiayen Lin, Dingcheng Shen, Gengwen Huang, Shuai Zhu, Lu Chen

**Affiliations:** 1 Division of Pancreatic Surgery Xiangya Hospital Central South University Changsha 410008 China; 2 Department of General Surgery Xiangya Hospital Central South University Changsha 410008 China; 3 National Clinical Research Center for Geriatric Disorders Xiangya Hospital Central South University Changsha 410008 China

Acute pancreatitis arises from the activation of digestive enzymes in pancreatic acinar cells, leading to autodigestion of the pancreas and surrounding tissues. It is a common digestive tract emergency which requires hospitalization, and its incidence is increasing worldwide [1]. In the past decade, several advances have been made in the treatment of acute pancreatitis [2]. However, there is still a lack of efficacious drugs for clinical practice, and the limited value of existing biomarkers for early warning of the severity of acute pancreatitis is a major obstacle. Thus, there is an urgent need to gain a better understanding of the molecular mechanisms of acute pancreatitis.

Circular RNAs (circRNAs) are a unique class of RNA molecules that are covalently closed [3]. Ongoing investigations have provided evidence that circRNAs govern downstream target expression by acting as miRNA sponges, functioning as transcription factors, interacting with RNA-binding proteins, and regulating alternative splicing [4]. These mechanisms support the pivotal role of circRNAs in a wide variety of physiological and pathological conditions, such as innate immunity, inflammation, neuronal function, and tumorigenesis [5].

**Figure 1:**
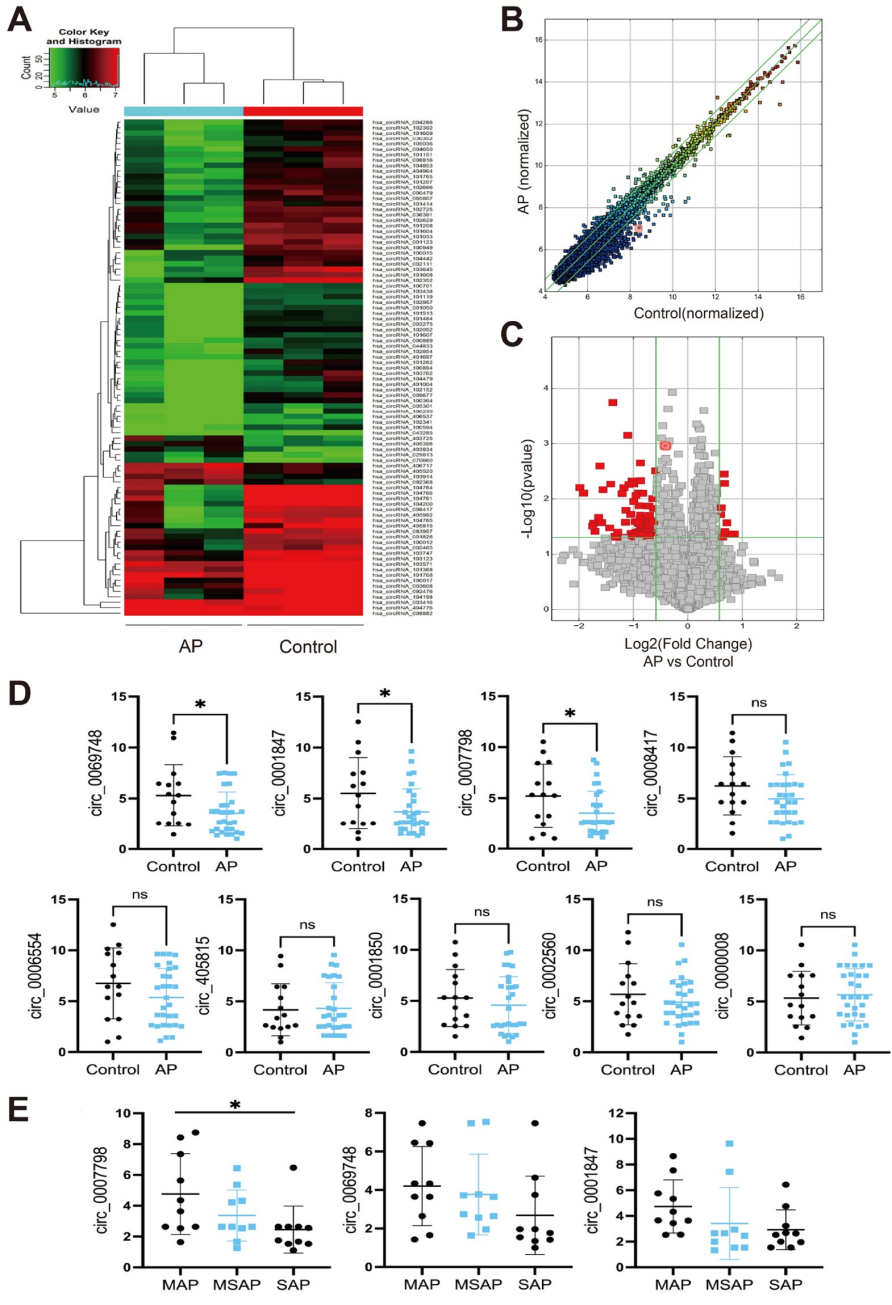
Figure 1 Identification and validation of differentially expressed circRNAs by microarray (A) Clustered heatmap of the differentially expressed circRNAs in the blood of three acute pancreatitis patients and three matched healthy individuals. (B) Scatter plot of circRNA expression between acute pancreatitis patients and healthy controls. The values of the X and Y axes are the normalized signal values or the averaged normalized signal values of groups of samples (log2 scaled). The green lines are the fold change lines. The circRNAs above the top green line and below the bottom green line showed more than a 1.5-fold change in expression between the two groups. (C) Volcano plots indicating the expression profiles of circRNAs in the two groups, and the red points in the plot represent the differentially expressed circRNAs with statistical significance. The values on the X-and Y-axes are the fold change values and P values, respectively. (D) The expression levels of 9 circRNAs were detected by RT-qPCR in fifteen healthy controls (controls) and thirty acute pancreatitis patients (AP). (E) The expression levels of 3 circRNAs were detected by RT-qPCR in acute pancreatitis patients with different disease severities (mild, MAP; moderate, MSAP; severe, SAP).

To explore the role of circular RNA in acute pancreatitis, we employed circRNA microarray technology (Arraystar Human circRNA Array V2) to examine the circRNA expression profile in the blood of three acute pancreatitis patients and three healthy controls. Clinical acute pancreatitis samples were obtained from Xiangya Hospital, Central South University. This study was approved by the Ethics Committee of Xiangya hospital (No. 2019010008). Normal control patients were recruited from among individuals who had visited Xiangya Hospital for a routine checkup. Written informed consent was obtained from all participants or their legal representatives for publication of data. The diagnosis and severity classification of acute pancreatitis were performed according to the American Gastroenterological Association guidelines and the Revised Atlanta Classification (RAC) [6]. circRNAs with a fold change ≥ 1.5 and a *P* value<0.05 were considered to be differentially expressed. As shown in Figure 1A, the two groups presented different expression profiles. We found that 91 circRNAs were significantly differentially expressed in the blood of acute pancreatitis patients, with 10 circRNAs exhibiting increased expression and 81 exhibiting decreased expression ( Figure 1B,C).

Among the differentially expressed circRNAs in acute pancreatitis, downregulated circRNAs are more prevalent than upregulated circRNAs, and the differential expression is more significant. Therefore, the present study focused on downregulated circRNAs. We selected circRNAs that are downregulated at least 2.5-fold and excluded those with fewer than 1000 bases to ensure the accuracy of qPCR. Based on these criteria, we identified nine circRNAs (circ_0006554, circ_0007798, circRNA_405815, circ_0001847, circ_0069748, circ_0001850, circ_0008417, circ_0002560, and circ_0000008) for validation by qPCR (Applied Biosystems, Foster City, USA) in blood samples from 30 acute pancreatitis patients (10 patients each with mild acute pancreatitis, moderate severe acute pancreatitis, and severe acute pancreatitis) and 15 healthy individuals. The levels of circ_0007798, circ_0001847, and circ_0069748 were significantly lower in acute pancreatitis patients than in normal controls, while the remaining circRNAs were not significantly differentially expressed ( Figure 1D). In addition, the levels of circ_0007798 increased gradually with the severity of acute pancreatitis, suggesting that circ_0007798 is associated with the clinical severity of the disease ( Figure 1E). Differential circRNAs have been studied for the diagnosis of pancreatic diseases [7]. The expression level of circ_0007798 can be used to grade the severity of acute pancreatitis and provide individualized treatment. Furthermore, homology analysis (NCBI blast) revealed that circ_0007798 has a high degree of conservation between rats and humans according to the basic local alignment search tool. In conclusion, according to the ranking order in terms of the integrated factors, circ_0007798, named circMAP3K5 because its gene symbol is *MAP3K5*, was finally chosen as the target for subsequent experiments.

**Figure 2:**
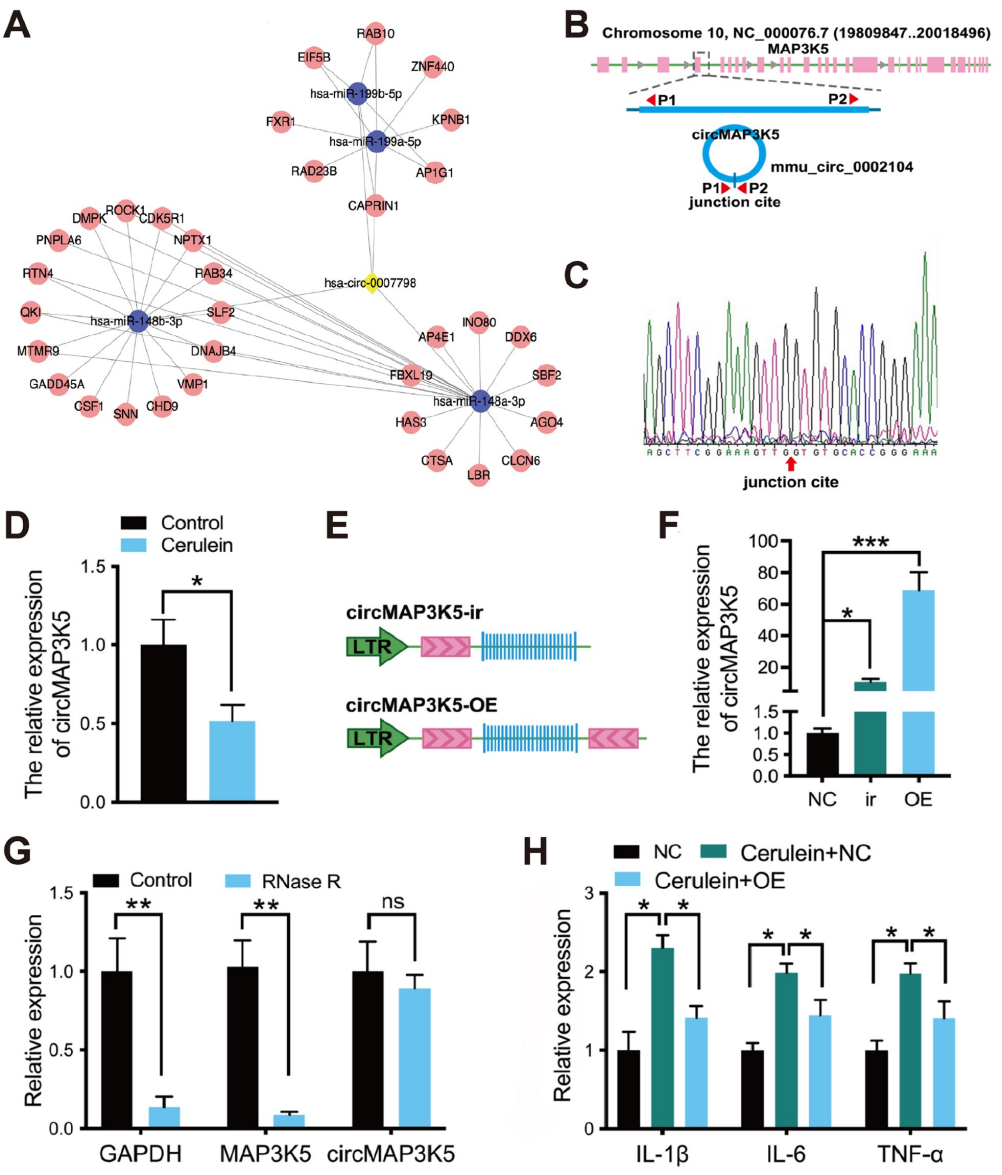
Figure 2 circMAP3K5 inhibits inflammation in acute pancreatitis (A) Annotation of the detailed regulatory relationships between circ-0007798 and target miRNAs predicted by TargetScan and miRanda. (B) Schematic illustration of circMAP3K5. The divergent primers P1/P2 were designed for the amplification of the circular form. (C) Sanger sequencing revealed that the RT-qPCR amplification products crossed the head-jail junction site. (D) The expression level of circMAP3K5 was detected by RT-qPCR in an acute pancreatitis model of MPC-83 induced by cerulein. (E) The sequence of circMAP3K5 was inserted into a lentiviral vector along with an endogenous upstream flanking genomic sequence (circMAP3K5-ir). Subsequently, the upstream flanking sequence was inserted downstream in an inverted orientation (circMAP3K5-OE). (F) The expression level of circMAP3K5 was detected by RT-qPCR after MPC-83 cells were transfected with empty lentiviral vector (NC), circMAP3K5-ir, or circMAP3K5-OE. (G) The expressions of GAPDH, MAP3K5 mRNA, and circMAP3K5 were analyzed by qRT-PCR using RNA from MPC-83 cells treated with RNase R. (H) The expressions of inflammatory cytokines (IL-1β, IL-6, and TNF-α) were detected by ELISA after MPC-83 cells treated with cerulein were transfected with circMAP3K5-OE or empty lentiviral vector (NC).

The current consensus is that circRNAs can function as miRNA sponges by competitively binding with miRNAs to modulate the expressions of target genes. To elucidate the potential sponge function of circMAP3K5 in acute pancreatitis, a circRNA-miRNA-mRNA interaction network was constructed using TargetScan and miRanda software analysis. Intersection analysis revealed that miR-148a-3p, miR-148b-3p, miR-199a-5p, and miR-199b-5p have the greatest potential for binding to circMAP3K5. The ceRNA network map further revealed the potential downstream targets of these miRNAs that could be regulated by circMAP3K5. In total, the network included 4 miRNAs and 34 mRNAs ( Figure 2A). Cai *et al*. [8] showed that miR-148a-3p inhibition inhibits necrosis by regulating PTEN in acute pancreatitis. Su *et al*. [9] reported that miR-199a-5p is significantly associated with the regulation of autophagy signaling in acute pancreatitis. Collectively, these findings expand our understanding of the potential functions of circMAP3K5 in the pathological processes of acute pancreatitis.

To further elucidate the biological function of circMAP3K5 in acute pancreatitis, we utilized the well-established cerulein-induced cell model of acute pancreatitis in the mouse pancreatic acinar cell line MPC-83 (the Cell Bank of the Chinese Academy of Sciences, Shanghai, China). circMAP3K5, transcribed from the *MAP3K5* gene, was amplified using divergent primer pair P1/P2 (P1, 5′-TTTGGACTCTAATTTCACGGACA-3′ and P2, 5′-TCCACCACCG CAATATCAAGA-3′), and a schematic illustration of circMAP3K5 is presented in Figure 2B. Sanger sequencing (GenePharma, Shanghai, China) confirmed that a head-to-tail junction site is present in the qPCR product of circMAP3K5 ( Figure 2C). As expected, the expression of circMAP3K5 is downregulated in the acute pancreatitis model of MPC-83 ( Figure 2D). Compared with the linear MAP3K5 and GAPDH mRNAs, circMAP3K5 was more resistant to RNase R digestion, suggesting that circMAP3K5 is more stable than linear MAP3K5 in MPC-83 cells ( Figure 2G).

To evaluate the role of circMAP3K5 in inflammation, a lentiviral vector-based system was constructed. The sequence of circMAP3K5 and the 1000 bases upstream of the transcription start site were inserted into the lentiviral vector (circMAP3K5-ir; Figure 2E), while the 1000 bases upstream of the transcription start site were inverted and inserted downstream (circMAP3K5-OE; Figure 2E) [10]. circMAP3K5-OE significantly increased the production of circ MAP3K5, while circMAP3K5-ir decreased the relative amount of circMAP3K5 ( Figure 2F). Overexpression of circMAP3K5 led to significant downregulation of IL-1β, IL-6, and TNF-α levels in the supernatant, indicating that circMAP3K5 can inhibit inflammation in acute pancreatitis ( Figure 2H).

In conclusion, our study provides an overview of differentially expressed circRNAs in acute pancreatitis and sheds light on their pathogenesis and molecular mechanism, as well as new targets for therapeutic intervention and prognostic prediction. Furthermore, we demonstrate that circMAP3K5 inhibits proinflammatory cytokine release, indicating its vital role in the pathological processes of acute pancreatitis.
